# European heart health survey 2019

**DOI:** 10.1002/clc.23478

**Published:** 2020-10-28

**Authors:** Luise Gaede, Marta Sitges, Johnson Neil, Eleonara Selvi, William Woan, Richard Derks, Helge Möllmann

**Affiliations:** ^1^ Erlangen‐Nürnberg Friedrich‐Alexander‐Universität Erlangen Nürnberg Erlangen Germany; ^2^ Institut Clínic Cardiovascular, Hospital Clínic Universitat de Barcelona Barcelona Spain; ^3^ Croi Heart and Stroke Charity and Global Heart Hub Galway Ireland; ^4^ Federanziani Senior Italia Rome Italy; ^5^ Heart Valve Voice Manchester UK; ^6^ Hart Volgers Amsterdam Netherlands; ^7^ Klinik für Innere Medizin I, St.‐Johannes‐Hospital Dortmund Germany; ^8^ Institut d'Investigacions Biomèdiques August Pi i Sunyer Barcelona Spain; ^9^ CIBERCV Instituto de Salud Carlos III (CB16/11/00354) Madrid Spain; ^10^ CERCA Programme/Generalitat de Catalunya Barcelona Spain

**Keywords:** aortic stenosis, awareness, heart valve disease, knowledge, survey

## Abstract

**Background:**

Rising life expectancy in the western population is increasing the prevalence of heart valve diseases (HVD).

**Hypothesis:**

The level of awareness and initial screening for HVD should be sufficient. The potential impact of HVD on the daily activities of the elderly population in Europe might affect our society.

**Methods and Results:**

A survey was conducted, including a total of 12 832 people aged ≥ 60 years in 11 European countries. Of all the people surveyed, 5.6% could correctly describe aortic valve stenosis. Most participants (75.0%) claimed they regularly do activities like sports or social activities, 29.2% provide care for a family member, friend or acquaintance. The majority (69.2%) would be prevented from doing these activities by symptoms such as chest pain, fatigue or shortness of breath. Having chest pain (76.5%) and shortness of breath (57.8%) were reasons for most people to arrange an appointment with their GP, whereas only 26.2% would visit a GP for fatigue. 67.6% of respondents claimed to be checked with a stethoscope by their GP occasionally, never, or only when they ask. The preferred treatment option for HVD is a keyhole procedure (45.8%), whereas open heart surgery would only be preferred by 7.0%.

**Conclusion:**

Knowledge about HVD is still low. Neither appointments with a GP driven by symptoms nor regular use of a stethoscope are a reliable guarantee for early diagnosis. With the over 60s in Europe playing an active role in social life, awareness campaigns and regular heart health checks may guarantee early diagnosis and treatment of HVD.

## 1 INTRODUCTION

Moderate or severe heart valve disease (HVD) occurs in over 13% of the population aged >75 years.^1^, ^2^ Due to the relationship between increasing life expectancy and degenerative HVD, the number of patients with severe HVD has been growing steadily over recent years^3^ and is likely to continue to grow. In the primary care setting, a significant proportion of patients with suspected heart failure are found to have HVD.^4^ The number of hospitalisations due to HVD doubled within the last 20 years and the proportion of people dying from HVD has continued to increase.^5^ The impact of HVD on survival holds especially true for severe aortic valve stenosis, which is the most common HVD in the elderly and affects up to 7% of the population older than 65 years.^1^ Untreated aortic stenosis is associated with a high mortality–even in patients without symptoms.^6^, ^7^, ^8^, ^9^ Nowadays, HVD can be treated successfully either with surgery or catheter‐based approaches s.^11^, ^12^, ^13^, ^14^ Therefore, to minimize mortality, patients with HVD should be diagnosed early, closely monitored and have access to timely and appropriate treatment.^10^ In order to achieve this, effective screening and detection, as well as a well‐informed population are the key. Prior Heart Health Surveys showed alarmingly low levels of awareness and knowledge of HVD.^14^, ^15^

On the other hand exponentially rising TAVI procedures have caused criticism. This is based on the assumption that patients are too old and too frail to profit from treatment.^16^ There has been much debate among clinicians as to whether some older patients would benefit from certain treatments, especially if they are elderly or too frail to receive open heart surgical interventions. Therefore, the 2019 European Heart Health Survey sought to reveal the impact of HVD symptoms on quality of life, daily activities, and societal contribution, to demonstrate why access to treatment is important. Additionally, the 2019 survey–as the previous surveys from 2017 and 2015–assessed again the awareness and concern about aortic stenosis and symptoms of heart valve disease as well as the frequency of stethoscope checks–a key step to the detection of the condition.

## 2 METHODS

### 2.1 Conduct of the survey

The survey was conducted in the following 11 European countries: Austria, Belgium, Germany, France, Ireland, Italy, Netherlands, Spain, Sweden, Switzerland and the United Kingdom.

An online survey was utilized for all territories. The online panels were actively managed and recruited for market research purposes. All participants went through a double opt‐in process and agreed to participate to provide honest opinions for market research studies. For each survey, an invitation was sent to respondents via email on a random basis within the target groups. Each invitation reiterated the terms and conditions for the research, including the potential use by the researchers of the information provided. Participants were reminded that personal identity and other personally identifiable details of the respondents would be protected. The survey was conducted in October 2019 and adheres to the code of conduct of the Market Research Society (MRS), the world's leading research association. It is ensured that all research is carried out in a professional and ethical manner. The survey is also based on the ESOMAR principles^17^ and fully compliant and in accordance with the Data Protection Act, the ICMJE Protection of Research Participants as well as with the Declaration of Helsinki. Explicit approval of an ethics committee was not necessary, as the data presented was derived from a survey based on voluntary participation linked with the agreement of the terms and conditions provided in the invitation.

### 2.2 Contents of the survey

The survey was designed and led by a European Steering Committee. The steering committee consisted of a mix of healthcare professionals and patient organizations. Patient representatives from Italy (Senior Italia), Ireland (Croi), the Netherlands (Hart Volgers) and the UK (Heart Valve Voice) were included in the steering committee. The Steering Committee led the development of questions and the design of the survey. All members are listed as authors.

The survey consisted of seven multiple choice questions and one open‐ended question. Please specify what you think aortic stenosis is. Answers to the open ended questions were matched against a set of key terms agreed by the Steering Committee. Answers consisting one or more of the key words was deemed correct.

Questions aimed to assess levels of awareness of aortic stenosis, general health concerns, and knowledge of HVD, but also to assess social and physical activity levels of the respondents, and frequency of stethoscope checks by general practitioners (GPs). Questions are displayed in Table 1. The survey consisted of seven multiple choice questions and one open‐ended question.

**TABLE 1:** Contents of the survey

Question	Potential answers	
Do you know what aortic stenosis is?	Yes, please specify	No
Which one of these diseases are you most concerned by?	Heart valve disease Cancer (eg, prostate, breast or lung cancer)	Stroke Respiratory disease Alzheimer's disease
Which of the following activities do you regularly do (on a weekly or monthly basis)?	Volunteering for a charity or community Playing sport/exercise Coaching/teaching Artistic/creative hobbies Community or local politics / governance	Social activities together with other elderly people Other I do not regularly do any activities
Do you provide care for a family member, friend or acquaintance?	Yes, I care for my partner Yes, I care for an elderly friend or acquaintance	Yes, I provide care for my grandchildren Yes, other No
Would chest pain, fatigue or shortness of breath prevent you from doing any of the listed activities? (tick all that apply)	Yes, caring for loved ones Yes, physical activity/activities Yes, sexual relations	Yes, hobbies and interests Yes, working/volunteering Yes, othe No *exclusive*
Which of the following symptoms would prompt you to seek an appointment with your GP?	Chest tightness/pain Abnormal heart beats: stronger, faster, slower, weaker, fluttering, irregular heartbeats Shortness of breath Feeling faint	Fatigue Reduced physical activity Feeling older than your age None of the above
When you visit a GP, how often do they check your heart with a stethoscope?	Every visit Every second visit Occasionally	Only when I ask for one Never
If you were to be treated for a heart condition, which one of these treatment options would you prefer?	Keyhole procedure that would cure you Lifelong daily drug treatment that would relive or better free you from symptoms	Lifelong weekly drug treatment that would relive or better free you from symptoms Open heart surgery that would cure you None of the above

The core questions (Q1, Q2, Q7) from previous surveys on awareness, concern and detection remained the same for 2019. Answers to the open ended questions (Q1: Please specify what you think aortic stenosis is?) were matched against a set of key terms agreed by the Steering Committee. Answers consisting one or more of the key words was deemed correct. The involvement of patient organizations led to the addition of questions focused on how over 60s contribute to the economy and play an important role in the society to help understand the potential impact of the condition on their lives and wider society. The survey was conducted in adherence to the STROBE guidelines.

### 2.3 Statistical analysis

Subgroup analyses were performed taking into consideration age (60‐64 years, 65‐69 years, 70‐74 years, 75‐79 years, 80+ years) and gender. Pearson chi‐squared test was performed for categorical variables with nominal scale. The alpha level of statistical significance was 0.05.

All data not displayed is available on request.

## 3 RESULTS

In total, 12 832 people aged 60 years or older in 11 European countries participated in the 2019 European Heart Valve Survey. The participants characteristics are listed in suppl Table 1.

*Q1. Do you know what “aortic stenosis” is?*

**FIGURE 1:**
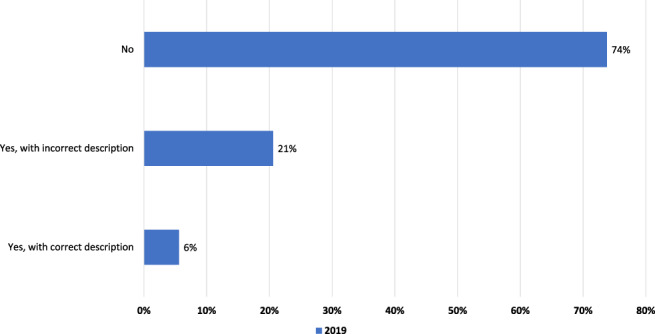
Q1. Do you know what “aortic stenosis” is?

In total, 26.2% (n = 3359; 2017:25.7%) of Europeans claimed to know what aortic stenosis is. However, only 5.6% of all respondents could correctly describe the condition (Figure 1). Women (6.4% vs 4.9%, *p* < 0.001), and younger people (6.4%: 60‐64 years vs 80+ years: 3.2%; *p* < 0.001) gave the correct description more often. People in the Netherlands (12.0%), Spain (8.6%) Italy (7.5%) Austria (7.3%) were more likely to know about aortic stenosis than people in Belgium (1.6%) and UK (2.4%); (*p* < 0.001).

*Q2. Which one of these diseases are you most concerned by?*

**FIGURE 2:**
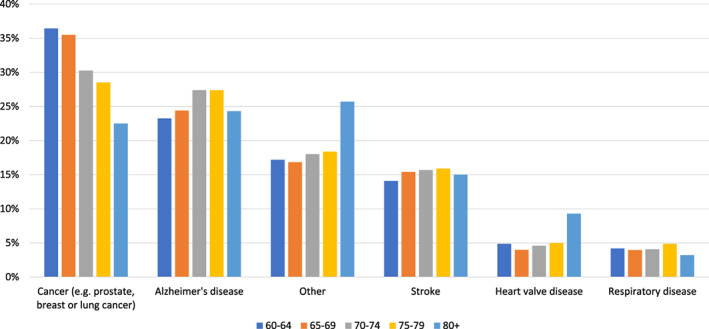
Q2: Which one of these diseases are you most concerned by? (by age)

The disease with the highest level of concern among the respondents is cancer (34.0%), followed by Alzheimer's Disease (24.8%) and stroke (15.0%). Only 4.6% of the respondents were most concerned about HVD. Male respondents were most concerned about cancer (36.8% vs 30.5%; *p* < 0.001), whereas female respondents were most concerned about Alzheimer's (29.8% vs 20.8%; *p* < 0.001). While concern about cancer decreased with age (60‐64 years: 36.4% vs >80 years: 22.5% *p* < 0.001), concern about HVD increased with age (60‐64 years: 4.9% vs >80 years: 9.3%; *p* < 0.001; Figure 2).

*Q3: Which of the following activities do you regularly do (on a weekly or monthly basis)?*

**FIGURE 3:**
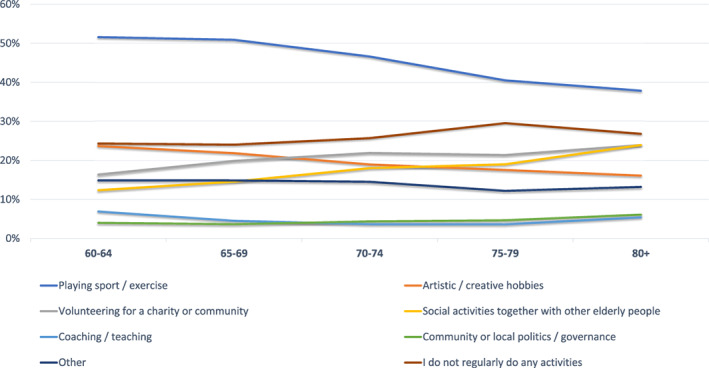
Q3: Which of the following activities do you regularly do (on a weekly or monthly basis)? (by age)

Three quarters of Europeans (75.0%) over 60 years regularly participate in activities on a weekly or monthly basis. The over 60s in Europe frequently engage in the following: 49.2% play sport or exercise, 21.5% have artistic or creative hobbies, 19.2% volunteer for a charity or a community, 15.0% do social activities together with other elderly people, 5.2% coach or teach, and 4.1% are involved with community or local politics. One quarter (25.0%) said they do not regularly do any activities. Pursuing exercise or sports (60‐64 years: 51.6% vs 80+ years: 37.9%; *p* < 0.001), as well as artistic hobbies (60‐64 years: 23.7% vs 80+ years: 16.1%; *p* < 0.001) decreases with age, whereas volunteering for charity or community (60‐64 years 16.4% vs 80+ years 23.9%, *p* < 0.001), social activities with other elderly people (60‐64 years: 12.4% vs 80+ years: 24.6%, *p* < 0.001) and being involved in politics (60‐64 years: 4.0% vs 80+ years: 6.1%; *p* < 0.001) increases significantly as people age (Figure 3).

*Q4: Do you provide care for a family member, friend or acquaintance?*

A third (29.2%) of Europeans over 60 years provide care for a partner, grandchild, friend or acquaintance or someone else. Women (32.9%) are more likely to care for others than men (26.3%; *p* < 0.001). The most common form of caregiving is to care for a partner (13.9%), followed by caring for someone else (8.9%) and for grandchildren (7.8%). Rarely, people care for a friend or acquaintance (3.8%). There is only a small age difference among carers when comparing the different age groups. One third of people aged 80 and above regularly care for other people (suppl Figure 1).

Q5: *Would chest pain, fatigue or shortness of breath prevent you from doing any of the listed activities? (Tick all that apply)*.

In total, 69.2% of over 60s claim that the symptoms of HVD (chest pain, fatigue or shortness of breath) would prevent them from doing certain activities. The majority (56.5%) said they would be prevented from doing physical activities, 23.7% would be prevented from working or volunteering, and 22.4% from pursuing hobbies and interests. Only 18.4% would avoid sexual relations and 18.1% would be prevented from caring for loved ones. A third (30.8%) claim the symptoms would not prevent them from doing any of the activities listed in the survey. Younger respondents are more likely to be prevented from activities due to symptoms of HVD that older respondents (60‐64 years: 70.9% vs 80+ years: 62.9%; *p* < 0.001).

*Q6: Which of the following symptoms would prompt you to seek an appointment with your general practitioner (GP)?*

In total, 93.7% of all respondents claim that having at least one of the symptoms of HVD would make them seek an appointment with their GP. Of the respondents, 76.5% would go to a GP if they experienced chest tightness/pain, 64.1% for abnormal heart beats, 57.8% for shortness of breath and 45.9% when feeling faint. Fewer people would visit a GP if they experienced symptoms not thought to be directly related to the heart, such as fatigue (26.2%), reduced physical activity (19.9%) and feeling older than their age (12.5%). Compared with younger people, elderly people stated more often that none of the symptoms would prompt them to see their GP (80+ years: 10.0%; 60‐64 years: 6.9%).

*Q7. When you visit a GP how often do they check your heart with a stethoscope?*

Most respondents claim they occasionally (35.4%) receive a stethoscope check, while only a third (28.2%) say that their GP checks their heart at every visit. 67.8% of the respondents get checked occasionally or more over this period.

Males (31.3%) are more likely to be checked at every visit compared to females (24.2%, *p* < 0.001)–a trend consistent throughout the 3 years (2017: males 30.2%, females 24.6%) the survey was conducted.

**FIGURE 4:**
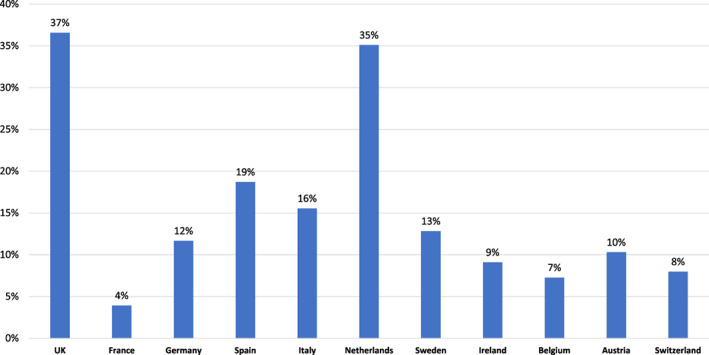
Q7: When you visit a GP, how often do they check your heart with a stethoscope (by country)

There was no significant difference in stethoscope use based on age. To check for differences in healthcare systems, stethoscope checks were analyzed in each participating country. Stethoscope checks for over 60s in Europe are highest in France at “every GP visit” (76.1%) and in checks received occasionally or more (92.8%). Belgium was second (56.9%, 82.2%) and lowest in the Netherlands (4.9%, 30%) and the UK (6.4%, 46.6%). In the UK, 36.6% of respondents claim to be never checked with the stethoscope, as did 35.1% of respondents in the Netherlands. However, only 3.9% of over 60s in France say they are never checked with a stethoscope (Figure 4).

*Q8: If you were to be treated for a heart condition, which one of these treatment options would you prefer?*

Europeans claim they would prefer a keyhole procedure (45.8%) compared to lifelong daily drug treatment (19.7%) or lifelong weekly drug treatment (19.2%). Open heart surgery is the least popular treatment option (7.0%) among over 60s in Europe (suppl Figure 2).

Men (45.5%) and women (46.2%) had very similar levels of preference for a keyhole procedure. Respondents aged 80 years and above were more likely to prefer daily drug treatments (25.4%) than younger respondents (60‐64 years: 18.4%; *p* < 0.001), and less likely to prefer open heart surgery (5.4% vs 6.4%, *p* = 0.15) or keyhole procedure (40.4% vs 45.5%, *p* < 0.001).

## 4 DISCUSSION

The 2019 European Heart Health Survey examined not only awareness levels of HVD, but also the daily activities and societal contribution of people aged 60 years and older throughout Europe. It additionally assessed: concerns about the symptoms of HVD, the frequency of stethoscope checks and the preferred HVD treatment options.

The results of this survey of over 12 000 people showed the following:

1. HVD is still not well known and has low levels of concern compared to other diseases such as cancer and Alzheimer's disease.
2. At the age when HVD typically develops, people are still very active and often contribute substantially to society by providing care to others.
3. Common symptoms of HVD lead to a GP visit for the majority of respondents, while atypical symptoms such as fatigue, reduced physical activity and feeling older than your age do not.
4. People aged 60 years and older are not regularly checked with a stethoscope by their GP
5. People over 60 years prefer to be treated with a keyhole procedure in the case of developing severe HVD, over open‐heart surgery and medications.

Despite the growing number of patients with HVD^12^, the number of people knowing and being concerned about HVD is still alarmingly low. Compared with previous surveys, there was only a small increase in people correctly describing aortic valve stenosis (2017:3.8%, 2019:5.6%), and a small increase in concern about HVD.^14^, ^15^

The percentage of people correctly describing aortic stenosis is close to the estimated prevalence of aortic stenosis in the population participating in the survey. The small increase might be due to higher prevalence of HVD in an aging population, and therefore respondents' friends, family, or even themselves may have been affected, or through the awareness campaigns started within the last years. Interestingly, the percentage of describing aortic stenosis correctly differed widely between the different countries in Europe. Whereas participants from the Netherlands gave the correct description in 12.0%, only 1.6% of the participants from Belgium gave the correct answer. Maybe the different healthcare systems or might have an influence on the level of knowledge.

The concern about morbidities differs between the age groups. Whereas young patients are more concerned about cancer, elderly patients are more concerned about heart valve disease, Alzheimer or other morbidities, which were not specified in more detail and could contain neurological as well as all other diseases including myocardial infarction. We show in our results, that the attitude towards morbidity and death certainly changes with age, especially regarding cancer. Elderly people might be more concerned about diseases baring the risk of neurological impairment or worse prognosis.

The survey results also show that elderly people nowadays are likely to be highly involved in social activities and play an important role in caring for others. If these people develop symptoms of HVD, the results suggest they will be prevented from fulfilling their normal everyday activities. This would have an impact on social networks and communities. Thus, detecting people with HVD and treating them early is very important.

Even if patients suffer from symptoms preventing them from doing their regular activities, they will not automatically seek an appointment with their GP. With typical symptoms like chest pain, three out of four would seek an appointment, but with atypical symptoms like reduced physical activity or fatigue only a minority would see their GP. Therefore, a significant proportion of patients with HVD do not a seek medical consultation and are consequently diagnosed late or never. The EU Heart Health Survey has demonstrated that around 50% of the patients undergoing surgery for HVD were in NYHA class III or IV, which unnecessarily increases postoperative complications and mortality.^18^ Early detection and treatment is associated with improved long‐term outcome in symptomatic patients.^10^ Additionally, patients with asymptomatic HVD might profit from early treatment as well.^9^ To overcome this, regular checks with a stethoscope could be an adequate detection method and should be performed. However, as of today only 28.2% of people over the age of 60 throughout Europe claim to be checked with a stethoscope at every visit. The lower number of women who are regularly checked compared to men is also alarming. There are substantial differences in rates of stethoscope check throughout the European countries surveyed. Whereas France continues to have a high percentage of people regularly getting checked with stethoscopes, the Netherlands and the UK show extremely low rates of stethoscope checks.

In recent years, treatment of HVD (in particular, the treatment of aortic valve stenosis) has been revolutionized. Randomized trials and big registries have shown positive outcome results of transcatheter aortic valve implantation (TAVI) compared to medical and surgical treatment‐ independent of the individual surgical risk.^11^, ^12^, ^13^, ^19^, ^20^ Additionally, TAVI offers advantages to the patient including shorter recovery time allowing patients to leave the hospital earlier and get back to normal life, which can lead to cost savings to healthcare systems.^21^ Discussions about long‐term durability of keyhole devices are still ongoing, but show promising results with no inferiority to surgical devices until now.^22^, ^23^, ^24^ As available data suggests TAVI as the gold standard for most of the patients with aortic valve stenosis, the numbers of TAVI procedures already exceeded the number of surgical aortic valve repair in many European countries. In Germany TAVI is performed already twice as often as isolated surgical aortic valve repair.^11^, ^12^ In case of other HVD than aortic valve stenosis, the results of keyhole procedures are promising and new techniques and devices may offer valuable treatment options.^25^, ^26^, ^27^, ^28^ This survey proves that people would definitely prefer keyhole procedures over surgery for the treatment of HVD, but also over medical daily or even weekly treatment. Despite this preference, the HVD survey 2017 showed that only a minority (19%) of respondents are aware of the possibility of receiving a keyhole procedure to treat HVD, whereas the majority are more likely to know about open heart valve replacement.^15^ People should be encouraged to seek for an appointment with their GP in case of HVD symptoms. People might be willing to undergo a curative therapy earlier and would seek a medical appointment earlier if it was better known that there a less invasive keyhole therapy was more widely available. Awareness campaigns, patient organizations and education on the different treatment options could help with this.

### 4.1 Limitation of the survey

The 2019 European Heart Health Survey was only fielded in countries in Western Europe. Respondents were pre‐selected based on their agreement and ability to participate in an online survey. Among other factors this might have influenced the age distribution with fewest participants in the age group >80 years. Nonetheless, the large number of respondents may overcome the latter limitations.

## 5 CONCLUSION

Concern and knowledge of HVD have increased over the years but remain low. Typical symptoms of HVD lead to a GP appointment for the majority of patients. However, for some patients with typical or atypical symptoms, a HVD diagnosis may still be missed due to low rates of regular stethoscope checks. The aging population in Europe means that people aged 60 years and above are likely to continue playing an active role in society and caring for others. The survey results show that the symptoms of HVD can prevent these people from taking part in these positive activities, suggesting that there may be a secondary negative consequence of HVD to society if it is not properly treated in time. Awareness campaigns should be advanced to draw attention to the wider impact on society, while continuing to raise awareness of the symptoms and treatment options of HVD to achieve a fully informed population. In addition, advocating for regular heart health checks for over 60s may help to guarantee early diagnosis of HVD in all patients.

## CONFLICT OF INTEREST

L. Gaede is consultant and receive speaker's honoraria from Abbott Vascular and Edwards Lifesciences. H. Möllmann has received speaker's honoraria from Edwards Lifesciences, and proctor and speaker's honoraria from Abbott Vascular and Boston Scientific. M Sitges has received speaker and consultant honoraria from Abbott Vascular, Medtronic and Edwards Lifesciences.

## AUTHOR CONTRIBUTIONS

Luise Gaede, Helge Möllmann interpreted the data and drafted the first manuscript. Marta Sitges, Johnson Neil, Eleonara Selvi, William Woan and Richard Derks helped in finishing and redaction of the manuscript.

## Supporting information


**Figure 1** Q4: Do you provide care for a family member, friend or acquaintance? (by age

**Figure 2** Q8: If you were to be treated for a heart condition, which one of these treatment options would you prefer? (% of total respondents)

**Table S1** Baseline characteristics

## Data Availability

Data available on request due to privacy/ethical restrictions.
