# Erratum

**DOI:** 10.1002/clc.23587

**Published:** 2021-03-13

**Erratum to “European heart health survey 2019”**

Gaede L, Sitges M, Neil J, Selvi E, Woan W, Derks R, Möllmann H. European heart health survey 2019. Clin Cardiol. 2020 Dec;43(12):1539–1546. doi: 10.1002/clc.23478. Epub 2020 Oct 28. PMID: 33111998; PMCID: PMC7724240.

In the above published article, the description of Figure 4 was published incorrectly.

The correct description of Figure 4 in page 5 should read:

**FIGURE 4:**
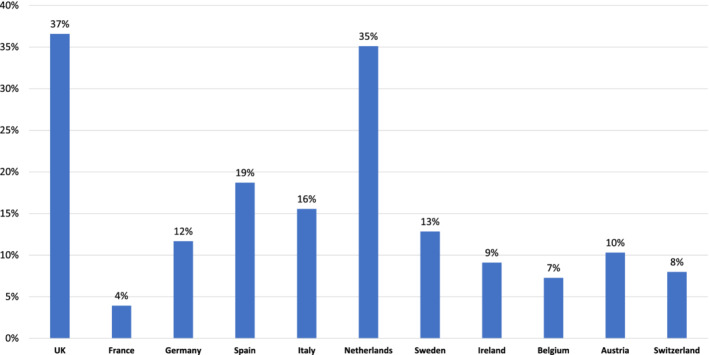
Q7: Percentage of people who said they were never checked with a stethoscope (by country)

We apologize for this error.

