# Supplementary figures and images for: European heart health survey 2019
Source: Clin Cardiol. 2020 Oct 28;43(12):1539–46. doi: 10.1002/clc.23478 (PMC7724240; doi:10.1002/clc.23478)

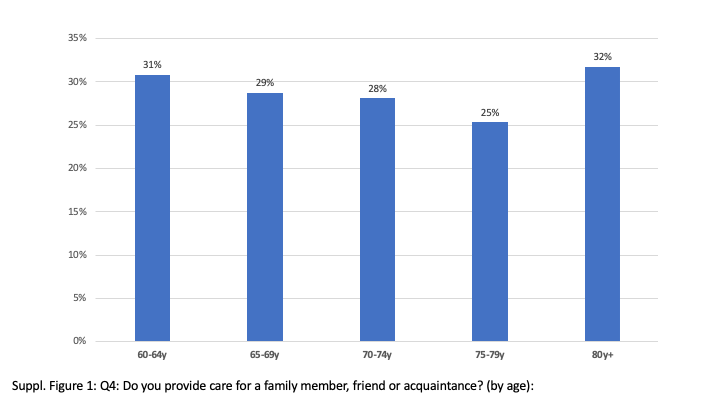

Supplement: Supplementary file 1 — Figure 1 Q4: Do you provide care for a family member, friend or acquaintance? (by age [file CLC-43-1539-s001.tiff]

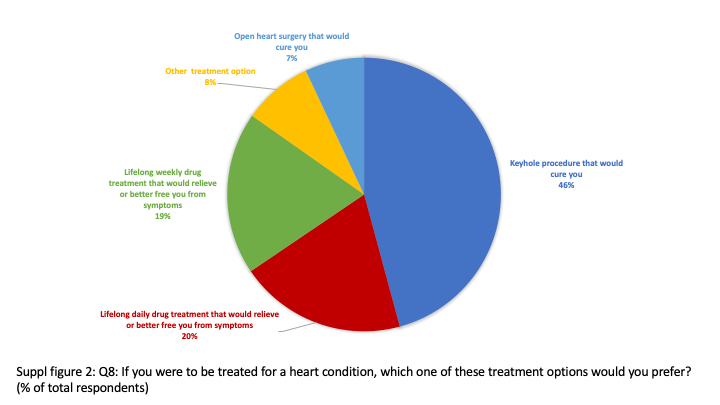

Supplement: Supplementary file 2 — Figure 2 Q8: If you were to be treated for a heart condition, which one of these treatment options would you prefer? (% of total respondents) [file CLC-43-1539-s002.tiff]
