# Supplementary material for: European heart health survey 2019
Source: Clin Cardiol. 2020 Oct 28;43(12):1539–46. doi: 10.1002/clc.23478 (PMC7724240; doi:10.1002/clc.23478)
Supplement: Supplementary file 3 — Table S1 Baseline characteristics [file CLC-43-1539-s003.docx]

Suppl Table 1: Participants Characteristics

|  | **N=12832** | % |
| --- | --- | --- |
| **Gender** | | |
| Male | 7,111 | 55.4% |
| Female | 5,721 | 44.6% |
| **Countries** | | |
| - Austria - Belgium - Germany - France - Ireland - Italy - Netherlands - Spain - Sweden - Switzerland - United Kingdom | 1,000  1,004  1,602  1,604  1,000  1,106  1,000  1,400  1,013  501  1,602 | 7.8%  7.8%  12.5%  12.5%  7.8%  8.6%  7.8%  10.9%  7.9%  3.9%  12.5% |
| **Age** | | |
| 60-64 | 4,814 | 37.5% |
| 65-69 | 4,112 | 32.0% |
| 70-74 | 2,658 | 20.7% |
| 75-79 | 968 | 7.5% |
| >=80 | 280 | 2.1% |
